# Relationships between motor scores and cognitive functioning in FMR1 female premutation X carriers indicate early involvement of cerebello-cerebral pathways

**DOI:** 10.1186/s40673-021-00138-0

**Published:** 2021-06-11

**Authors:** Elsdon Storey, Minh Q. Bui, Paige Stimpson, Flora Tassone, Anna Atkinson, Danuta Z. Loesch

**Affiliations:** 1 Department of Medicine (Neuroscience), Monash University, 5th Floor, Centre Block, Alfred Hospital Campus, Commercial Road, Melbourne, Victoria 3004 Australia; 2 Centre for Molecular, Environmental, Genetic and Analytic, Epidemiology, University of Melbourne, Parkville, Victoria Australia; 3 Wellness and Recovery Centre, Monash Medical Centre, Clayton, Victoria Australia; 4 Department of Biochemistry and Molecular Medicine, University of California, Davis, School of Medicine and M.I.N.D. Institute, University of California Davis Medical Center, Davis, California USA; 5 School of Psychology and Public Health, La Trobe University, Melbourne, Bundoora, Victoria Australia

**Keywords:** *FMR1* premutation, CGG repeats, Female premutation carriers, Tremor/ataxia scales, Cognitive tests, Motor-cognitive scores relationships, Fragile X-associated tremor ataxia

## Abstract

**Background:**

Smaller expansions of CGG trinucleotide repeats in the *FMR1* X-linked gene termed ‘premutation’ lead to a neurodegenerative disorder: Fragile X Associated Tremor/Ataxia Syndrome (FXTAS) in nearly half of aged carrier males, and 8–16% females. Core features include intention tremor, ataxia, and cognitive decline, and white matter lesions especially in cerebellar and periventricular locations. A ‘toxic’ role of elevated and expanded *FMR1* mRNA has been linked to the pathogenesis of this disorder. The emerging issue concerns the trajectory of the neurodegenerative changes: is the pathogenetic effect confined to overt clinical manifestations? Here we explore the relationships between motor and cognitive scale scores in a sample of 57 asymptomatic adult female premutation carriers of broad age range.

**Methods:**

Three motor scale scores (ICARS-for tremor/ataxia, UPDRS-for parkinsonism, and Clinical Tremor) were related to 11 cognitive tests using Spearman’s rank correlations. Robust regression, applied in relationships between all phenotypic measures, and genetic molecular and demographic data, identified age and educational levels as common correlates of these measures, which were then incorporated as confounders in correlation analysis.

**Results:**

Cognitive tests demonstrating significant correlations with motor scores were those assessing non-verbal reasoning on Matrix Reasoning (*p*-values from 0.006 to 0.011), and sequencing and alteration on Trails-B (*p*-values from 0.008 to 0.001). Those showing significant correlations with two motor scores-ICARS and Clinical Tremor- were psychomotor speed on Symbol Digit Modalities (*p*-values from 0.014 to 0.02) and working memory on Digit Span Backwards (*p*-values from 0.024 to 0.011).

**Conclusions:**

Subtle motor impairments correlating with cognitive, particularly executive, deficits may occur in female premutation carriers not meeting diagnostic criteria for FXTAS. This pattern of cognitive deficits is consistent with those seen in other cerebellar disorders. Our results provide evidence that more than one category of clinical manifestation reflecting cerebellar changes – motor and cognitive - may be simultaneously affected by premutation carriage across a broad age range in asymptomatic carriers.

**Supplementary Information:**

The online version contains supplementary material available at 10.1186/s40673-021-00138-0.

## Introduction

Large CGG repeat expansions (> 200 repeats) in the fragile X mental retardation 1 (*FMR1*) X-linked gene, labelled ‘full mutations’, cause the Fragile X syndrome (FXS), a neurodevelopmental disorder resulting from decreased FMR1 translation. Since this mutation is unstable across generations if transmitted through the female parent, the mothers of FXS children are usually carriers of smaller expansions ranging from 55 to 200 CGG repeats, labelled ‘premutations’. Yet smaller expansions (41 to 54 repeats), which do not expand into the full mutation range across two generations but which are nevertheless linked to neurodegenerative changes, are termed “grey zone” expansions [1, 2]. The population prevalence of *FMR1* premutations ranges from 1 in 130 to 1 in 250 females, and from 1 in 250 to 1 in 810 males [3, 4]. Both male and female premutation carriers may develop a progressive, late onset neurodegenerative condition termed Fragile X Associated Tremor/Ataxia Syndrome (FXTAS) as they age, with a much higher rate in males (between 40 and 50% after the age of 55) than in females in the same age group (approximately 15%) [5–8]. The core features of this syndrome are kinetic tremor and gait ataxia, with cognitive decline and elements of parkinsonism. The major changes identified on MR imaging are cerebral atrophy and white matter disease, the latter being most prominent in the middle cerebellar peduncles (‘MCP sign’), especially in affected males [9, 10], and in the splenium of the corpus callosum in either sex [11]. Most typical FXTAS neuropathological changes are widespread intranuclear inclusions abundant in neurones and astrocytes [12], extending to autonomic nervous and neuroendocrine systems and myocardial cells [13–15]. These inclusions are composed principally of ~ 200 proteins, with over half involved in RNA binding and/or protein turnover (reviewed in MA et al. 2019 [16]). Their mRNA contents [17] gave rise to suggested pathogenetic mechanisms of FXTAS involving toxic gain-of-function of the elevated expanded CGG-repeat mRNA [18–20], and reviewed in [21], whilst a contribution of the accumulation of toxic poly-glycine peptides to these mechanisms are still being debated [16, 22, 23].

The penetrance of FXTAS is reduced in female premutation carriers, which can be, at least partly, attributed to the protective effect of the normal *FMR1* allele on the second X chromosome [24]. However, despite this evident neuroprotective effect, female carriers may present with a number of other clinical changes, such as premature menopause- FXPOI -[25], fibromyalgia, autoimmune thyroid disease, as well as psychiatric problems, predominantly anxiety and depression [26–34].

Apart from general health and psychiatric issues, few studies have explored subtle impairments that might be directly related to brain changes in carrier PM females, in the absence of overt neurological symptoms or signs. These studies, based on small samples, demonstrated deficits on a range of tasks of executive functioning requiring rapid temporal responses [35], or subtle impairment of postural stability [36], compared with control non-carriers. These results suggested that there may be a slow subclinical decline in executive functioning and/or degradation of motor functioning, combined with (and perhaps augmented by) stress and diminished adaptive capacity, in apparently asymptomatic premutation females.

Here we have taken a novel approach to expand on this issue by exploring the relationships of three motor scale scores - for tremor/ataxia, parkinsonism, and tremor, respectively - with the results of a range of neurocognitive tests, in a sample of neurologically asymptomatic, apparently unaffected adult (mainly postmenopausal) females carrying small expansion *FMR1* alleles within the premutation range (53 cases) or the grey zone range (4 cases). We hypothesise that performance on these two broad clinical domains – motor and cognitive - will be correlated. If this is borne out, it will suggest an underlying pathological process simultaneously affecting motor and cognitive performance at a sub-symptomatic level, and linked to premutation carriage across a broad range of age and CGG small expansion sizes.

## Materials and methods

### Subjects

The results of this study are based on retrospective analysis of 57 adult females (including 53 premutation and 4 grey zone carriers), who were originally ascertained through cascade testing of the large cohort of fragile X families described in our earlier publications [37–43], and who participated in the 2001–2003 project supported by research grants from the National Health and Medical Research of Australia (NHMRC) and the National Institute of Health, US, to DZL & ES. Except for one East Asian, all participants were white Caucasian. They provided informed consent according to protocols approved by the La Trobe University and Monash University Human Research Ethics Committees.

The age of participants ranged from 26 to 85 (mean 51.5 years); the size of CGG expansion ranged from 40 to 54 (for four grey zone carriers) and from 55 to 175 (for 53 premutation carriers).. Mean FMR1 mRNA level was 1.44, ranging from 0.9 to 2.7. Mean activation ratio (assessed in a subgroup of 12 premutation carriers) was 0.56. The demographics of the sample are presented in Table 1 of the supplementary material. None of the female participants in this sample (classified as ‘unaffected’) were diagnosed with - or reported any symptoms of - FXTAS, or any serious health issues.

**Table 1:** Spearman’s rank correlation (ρ) between each motor score and psychological score, adjusted for age and/or year of education (whenever appropriate)

		UPDRS		Clinical Tremor		ICARS Total	
	N	ρ	*p-*value	ρ	*p-*value	ρ	*p-*value
Vocab SS	48	−0.07	0.650	− 0.24	0.099	− 0.07	0.658
MR SS	49	−0.36	0.011*	−0.36	0.011*	− 0.39	0.006*
DS Forwards	48	−0.07	0.634	−0.17	0.237	−0.20	0.185
DS Backwards	48	−0.29	0.049	−0.33	0.024*	− 0.37	0.011*
PRO-rated IQ	48	−0.33	0.023*	−0.38	0.008*	− 0.42	0.003*
TMT A (raw score)	46	0.21	0.168	0.53	< 0.001*	0.46	0.001*
TMT B (raw score)	46	0.51	< 0.001*	0.39	0.008*	0.50	< 0.001*
TMT B-A	46	0.43	0.003*	0.21	0.159	0.46	0.001*
HVLT-R DR (t-score)	47	−0.14	0.364	0.11	0.460	0.02	0.891
HVLT-R DRI (t-score)	47	0.01	0.981	−0.01	0.946	0.17	0.262
SDMT (raw score)	47	−0.28	0.060	−0.43	0.002*	−0.36	0.014*

*p-values remain < 0.05 after adjustment for multiple testing using False Discovery Rate

### Methods

#### Motor and cognitive scale scores

Structured medical history and standard neurological motor rating scales with established inter-rater reliabilities [44–46] were administered by two neurologists (ES & DZL) with relevant experience in these scales from previous studies. Motor rating scales consisted of the Unified Parkinson’s Disease Rating Scale Part III-Motor (UPDRS-III [47]), the International Cooperative Ataxia Rating Scale (ICARS [48]), and the Clinical Rating Scale for Tremor [49].

The Vocabulary and Matrix Reasoning subtests of the Wechsler Adult Intelligence Scale (Third Edition; WAIS-III) were used to calculate a prorated Full-Scale IQ score [50], with Matrix Reasoning providing a measure of non-verbal reasoning. WAIS-III Digit Span component (forward and backward separately) were employed as measures of attention and working memory, respectively [50]. Executive functioning was also assessed using a measure of divided attention/set shifting, the Trail Making Test (TMT) [51]. The Symbol Digit Modalities Test was used as a measure of psychomotor processing speed [52]. The Hopkins Verbal Learning Test-Revised, (HVLT-R) [53] is a standard measure of verbal anterograde episodic learning. The HVLT-R delayed recall and discrimination recognition indices were employed as measures of recall and recognition memory, respectively.

#### Genetic molecular measures

*All assays were conducted at the MIND Institute, University of California Davis Medical Center, Sacramento, CA, USA.*

##### CGG sizing

Genomic DNA was isolated from peripheral blood lymphocytes using standard methods (Purygene Kit; Gentra,Inc., Minneapolis, MN). For Southern blot analysis, 10 micrograms (μg) of isolated DNA was digested with EcoRI and NruI. Hybridization was performed using the specific FMR1 genomic dig labelled StB12.3 probe as previously described [54]. Genomic DNA was also amplified by PCR [55].

##### FMR1 mRNA expression level measurements

Total RNA was isolated from 3 mL of blood collected in Tempus tubes (Applied Biosystems, Foster City, California, USA). The measurement of FMR1 mRNA expression levels was carried out by quantitative Real Time qRT-PCR using custom-designed Taqman gene expression assays (Applied Biosystems) as previously described [18].

##### Activation ratio

Activation ratio (AR) indicates the proportion of cells that carry the normal allele on the active X chromosome, so that AR = 1.00 indicates a normal allele active in 100% of the cells. It was measured based on the intensity of the appropriate bands on Southern blots as described in Tassone et al. [56] .

### Statistical analysis

Summary statistics for sample characteristics, cognitive and motor scores, are presented by both mean and standard deviation (SD), and median and interquartile (IQR). Distribution of age, CGG repeat size and motor scores were estimated using the Kernel density method. The relationship between each of the phenotypic scores (as outcome), and CGG repeat size, FMR1 mRNA, age and the level of education (as predictors), was assessed using robust regression. This method down-weights the effect of outliers and influential observations when present. If a relationship was significant, adjustment was made for these predictors by using the residual score (the difference between actual and predicted score) to assess relationships among these outcomes, using Spearman’s rank correlation. False Discovery Rate (FDR) was used to adjust for multiple testing. All analyses were carried using commercial software STATA, version 16 (*http://www.stata.com*).

## Results

**Fig. 1:**
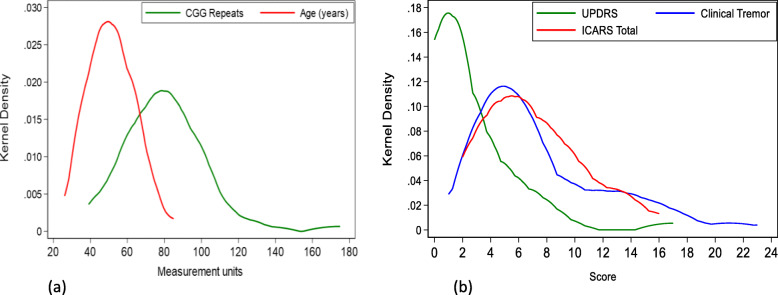
Kernel density distribution of age and CGG repeat size (**a**) and three motor scores (**b**) in the total sample of female carriers

The descriptive statistics (in supplementary Table 1) include all the core measures used in the correlations presented in Table 1. There was a single individual aged 26, and about one third of the participants were aged between 35 and 50, but, as shown in Fig. 1a, the majority was > 50 years of age. CGG repeat sizes ranged from 40 to 180, with the mode at approximately 80. A somewhat unexpected result concerned all three motor scales (Fig. 1b), where one individual (out of 49) scored 17 on the UPDRS; one scored 23 and ten others scored between 11 and 15 on the Clinical Tremor Scale, and nine individuals scored between 11 and 16 on the ICARS. Rare individuals scored highly on more than one scale. The average scores for ICARS and UPDRS also appear elevated compared with normal control values from the literature for similar age groups but for sexes combined (mean 4.07 ± 2.19, range 1–9 –for ICARS in: Fitzpatrick et al.,[57]; and mean 1.9 + − 2.0-for UPDRS in: Postuma et al., 2012 [58]). There is an absence of any control data for the Clinical Tremor Scale in the literature. It is obvious from the data in supplementary Table 1 that the mean values are towards the upper end of available control values, and the range for the ICARS are also increased relative to the normative values. We categorized this increase as sub-symptomatic since it had not generated any specific medical diagnoses or realization of abnormality on the part of those individuals presenting with evidently abnormal scores. Of 38 participants who underwent routine 1.5 Tesla T2 (FLAIR) MRI scanning, only one participant had possible subtle unilateral MCP sign, and five clearly had T2 hyperintensity in the splenium of corpus callosum. The wide range of scores is also noticeable for the cognitive test results.

The major focus of this study, however, was to ascertain if there is a systematic trend towards parallel motor and cognitive dysfunctions in apparently unaffected female premutation carriers across a wide age range. Firstly, using robust regression, we established that there were no significant relationships between any motor or cognitive scores and either the CGG repeat size or mRNA level (except one significant correlation of the latter with HVLT-R DR, which did not survive adjustment for FDR). However, a majority of both motor and cognitive scores was significantly related to age and level of education, which were subsequently included as confounders in correlations between the motor and cognitive scores.

The results of relationships between all three motor scores (UPDRS, Clinical Tremor and ICARS) and the 11 cognitive test scores are shown in Table 1. All three motor scores are significantly and consistently correlated with MR, DS Backwards, and TMT B, as well as with Pro-rated IQ (both for 2 - and 1-sided *p* values, and after FDR adjustment). Additionally, both Clinical Tremor and ICARS scores are highly correlated with SDMT. The ICARS score also correlated with TMT B-A.

Two aspects of these correlation results are of special interest: first, the cognitive measures showing significant relationships with the motor scores reflect aspects of psychomotor speed and executive functioning; second, that the largest number of motor x cognitive correlations concern the ICARS and Tremor scale scores, which represent the type of motor dysfunctions typically occurring in FXTAS.

## Discussion

This is the first study relating performance on three motor clinical scales - assessing tremor, ataxia and parkinsonism- to the level of cognitive functioning in a sample of non-FXTAS clinically asymptomatic adult female premutation carriers. We applied the ICARS and Clinical Tremor scales, which are suitable measures of motor deficits used for eliciting and rating cerebellar signs in tremor-ataxia disorders, including the FXTAS spectrum, where they highlight core neurological manifestations. Although the UPDRS scale used here largely reflects parkinsonian features, it overlaps with the other two motor scales, particularly as regards tremor, and thus shows significant correlations with the above scores in various cerebellar degenerative disorders [59]. The study results confirm our prediction that the broad clinical domain of tremor and ataxiawould be correlated with cognitive domain. As this study is cross-sectional rather than longitudinal, we can only infer from these correlations that early neurodegenerative changes involving cerebellar peduncles, simultaneously affect motor and cognitive performance at a sub-symptomatic but clinically detectable level. The observed relationships concern a set of motor and cognitive traits, which are reminiscent of deficits occurring in male or female FXTAS. We may therefore suggest that this postulated sub-symptomatic common underlying neuropathology may progress to a more extensive form of cerebellar white matter degeneration, with overt clinical manifestations of tremor/ataxia-FXTAS in a proportion of carriers included in our sample. It is of special interest that the cognitive correlates of motor scores identified in our study correspond to a set of deficits which has been reported to co-occur in various other conditions associated with lesions of the cerebello-thalamic and thalamo-cortical efferent, and cortico-pontine and ponto-cerebellar afferent tracts (see O’Halloran et al. [60] for review).

Although the association of the tremor-ataxia motor scales with cerebellar disorders has long been recognized, evidence for the relevance of the cerebellum to a wide range of selected cognitive functions has accumulated more recently, supported by functional magnetic resonance imaging (fMRI) and positron emission tomography (PET) findings in normal controls [61]. Apart from specific cognitive impairments (particularly affecting executive functions), cerebellar damage has been implicated in complex behavioural changes, such that ataxia, combined with multiple cognitive and behavioural impairments has been termed the Cerebellar Cognitive Affective Syndrome [62]. It is therefore likely that our results, showing correlations between all three motor scores and several cognitive scale scores, though occurring in asymptomatic, with minimal evidence of specific MRI changes, premutation carriers, may be a specific example of this syndrome in evolution from the sub-symptomatic, stage associated with microstructural changes in the cerebellar peduncles to clinically- and radiologically – apparent damage in FXTAS. It is of some relevance that Filley et al. [63], by applying advanced and sensitive MRI techniques - diffusion tensor imaging (DTI) and magnetic resonance spectroscopy (MRS)- have shown that microstructural white matter abnormalities in the middle cerebellar peduncles (MCP) and the genu and splenium of the corpus callosum (without visible T2 MCP sign) correlate with executive dysfunction and slowed processing speed in a sample of male premutation carriers without FXTAS. The findings of subtle changes in the integrity of white matter in premutation male carriers without, or prior to, the occurrence of FXTAS [64–66], and of abnormal trajectories in cerebellar and brain stem volumes from early adulthood in these carriers [67] has also provided direct evidence for preclinical brain changes. However, the possibility of similar changes in non-FXTAS premutation females remains relatively unexplored, except for important and relevant finding of the presence of abundant intranuclear inclusions - widespread throughout the brain and typical of FXTAS in both FXTAS and non-FXTAS female carriers [25].

Indeed, a broad range of cognitive deficits, including executive functioning, visuospatial processing, linguistic abilities and affective processes were features of these impairments in the original description [58], and in subsequent studies (O’Halloran et al. [60] see for review). Not all of these domains and subdomains were assessed here, and of those that were, not all showed correlation with the motor scales. However, those that correlated strongly with these scales were the only ones dependent on executive processing or psychomotor speed and execution.

We have not observed significant relationships between either the motor or the cognitive scores included in our analysis with either CGG repeat size or FMR1 mRNA levels. Earlier results have been inconsistent in this respect [68–70]. These varied results have been thought to be a consequence of small sample sizes impacting adversely an analytic power, particularly in the setting of the potentially weaker and more complex genetic effect on the phenotype of women with X-linked mutations. In addition, it is known that there is a brain-blood difference in *FMR1* mRNA expression, and potentially of the CGG expansion [17, 71–73] and of the activation ratio (AR) The latter did not show any relation to any of the cognitive or other phenotypic test scores in a recent comprehensive study [74], supported by our own (unpublished) data from the present sample. For this reason, activation ratio was not considered as a potential confounder in correlation analysis.

In conclusion, despite the limitations of the study, including relatively small sample size, and the lack of post-mortem neuropathological studies addressing the issue of microstructural damage to cerebellar peduncles in asymptomatic females, the results are important at two levels. First, they provide further evidence of cerebellar involvement in cognition, and especially in the multifaceted executive function domain tracking specific motor dysfunction. Importantly, we show that both motor and cognitive involvement can be detected in asymptomatic individuals. Second, they confirm that the two broad clinical domains -motor and cognitive-are intercorrelated, indicating that early neurodegenerative changes in the form of widespread disorders of the cerebellum simultaneously affect motor and higher cognitive performance at a sub-symptomatic but clinically detectable level in non-FXTAS adult premutation female carriers.

## Supplementary Information

**Additional file 1: Table S1.** Characteristics of sample.

## Data Availability

The datasets used and/or analysed during the current study are available from the corresponding author on reasonable request.
