# Supplementary material for: Relationships between motor scores and cognitive functioning in FMR1 female premutation X carriers indicate early involvement of cerebello-cerebral pathways
Source: Cerebellum Ataxias. 2021 Jun 11;8:15. doi: 10.1186/s40673-021-00138-0 (PMC8196444; doi:10.1186/s40673-021-00138-0)
Supplement: Supplementary file 1 — Additional file 1: Table S1. Characteristics of sample. [file 40673_2021_138_MOESM1_ESM.docx]

Supplementary material

**Supplementary Table 1: Characteristics of sample**

| Variable | N | Mean | SD | Median | IQR | Min-Max |
| --- | --- | --- | --- | --- | --- | --- |
| Characteristic |  |  |  |  |  |  |
| Age | 57 | 51.5 | 11.6 | 52.0 | 16.0 | 26-85 |
| Age of menopause | 29 | 43.7 | 6.78 | 45.0 | 8.0 | 27-53 |
| Year of Education | 57 | 12.6 | 3.2 | 12.0 | 4.0 | 4-22 |
| CGG repeats | 57 | 80.3 | 23.1 | 77.0 | 24.0 | 41-175 |
| mRNA | 45 | 1.43 | 0.49 | 1.20 | 0.70 | 0.9-2.7 |
| Motor Score |  |  |  |  |  |  |
| UPDRS | 51 | 2.41 | 3.16 | 1.0 | 4.0 | 0-17 |
| Clinical Tremor | 52 | 7.48 | 4.73 | 5.0 | 5.5 | 1-23 |
| ICARS Total | 52 | 7.08 | 3.58 | 7.0 | 5.0 | 2-16 |
| Cognitive measures |  |  |  |  |  |  |
| Vocab SS | 57 | 9.83 | 2.59 | 10.0 | 3.00 | 2-17 |
| MR SS | 57 | 11.4 | 3.29 | 12.0 | 3.00 | 2-17 |
| DS Forwards | 57 | 9.77 | 2.28 | 10.0 | 3.00 | 5-15 |
| DS Backwards | 57 | 6.50 | 2.26 | 6.00 | 3.00 | 3-14 |
| Pro-rated IQ | 57 | 104 | 14.0 | 106 | 15.5 | 54-137 |
| TMT A (raw score) | 52 | 36.9 | 17.2 | 35.0 | 11.5 | 17-114 |
| TMT B (raw score) | 52 | 83.2 | 39.1 | 73.0 | 42.0 | 37-255 |
| TMT B-A | 52 | 44.4 | 32.2 | 39.0 | 29.0 | 0-191 |
| HVLT-R DR (t-score) | 55 | 48.4 | 10.7 | 50.0 | 11.0 | 20-63 |
| HVLT-R DRI (t-score) | 55 | 50.8 | 9.25 | 52.0 | 13.0 | 20-60 |
| SDMT (raw score) | 58 | 48.9 | 11.8 | 49.0 | 14.0 | 18-74 |
| DASS (raw scores) |  |  |  |  |  |  |
| Stress | 56 | 9.32 | 7.90 | 8.0 | 10.0 | 0-32 |
| Anxiety | 56 | 4.54 | 5.76 | 2.0 | 6.0 | 0-28 |
| Depression | 56 | 4.68 | 5.46 | 2.0 | 7.0 | 0-22 |
| SCL90 GSI | 57 | 49.8 | 11.48 | 50.0 | 19.0 | 30-69 |

Max = maximum; Min = minimum; IQR = Interquartile range.

Vocab SS = WAIS-III Vocabulary Scaled Score; MR SS = WAIS-III Matrix Reasoning Scaled Score; DS Forwards = WAIS-III forwards digit span subtotal; DS Backwards = WAIS-III backwards digit span subtotal; TMT-A and -B = Trail-Making Test A and B; HVLT-R DR = Hopkins Verbal Learning Test-Revised, Delayed Recall; HVLT-R DRI = Hopkins Verbal Learning Test-Revised Delayed Recognition Index; SDMT = Symbol-Digit Modalities Test; DASS = Depression, Anxiety and Stress Scale; SCL90 Global Severity Index.
